# Geranylgeraniol (GGOH) as a Mevalonate Pathway Activator in the Rescue of Bone Cells Treated with Zoledronic Acid: An In Vitro Study

**DOI:** 10.1155/2019/4351327

**Published:** 2019-01-09

**Authors:** Riham M. Fliefel, Saleh A. Entekhabi, Michael Ehrenfeld, Sven Otto

**Affiliations:** ^1^Experimental Surgery and Regenerative Medicine (ExperiMed), Ludwig-Maximilians University, Munich 80336, Germany; ^2^Department of Oral and Maxillofacial Surgery, Ludwig-Maximilians University, Munich 80337, Germany; ^3^Department of Oral and Maxillofacial Surgery, Alexandria University, Alexandria 21500, Egypt

## Abstract

Bisphosphonates (BPs) are the keystone to treat bone disorders. Despite the great benefits of BPs, medication-related osteonecrosis of the jaw (MRONJ) arouse as a potential side effect. Nitrogen-containing BPs (N-BPs) as zoledronate (ZA) act by the inhibition of specific enzymes of the mevalonate pathway resulting in altering protein prenylation which is required for the posttranslational maturation of the small GTP-binding proteins. Geranylgeraniol (GGOH) is an intermediate product in the mevalonate pathway having positive effects on different cell types treated with BPs by salvaging protein prenylation improving cell viability and proliferation in tissue regeneration, thus overcoming N-BP-induced apoptosis. Here, the effect of different concentrations of zoledronate (ZA) on the bone cells has been investigated by cell viability assay, live/dead staining, and western blot to understand if GGOH was able to rescue bone cells and levels of statistical significance were indicated at ^∗^*P* < 0.05, ^∗∗^*P* < 0.01, ^∗∗∗^*P* < 0.001, and ^∗∗∗∗^*P* < 0.0001. Although the high concentration of ZA had significantly decreased the cell viability in the bone cells, GGOH reversed the action of ZA on the cells while at very high concentration; it caused severe reduction in the cell viability. Rap1A, a member of the GTPases family, was expressed in the negative controls but was absent in cells treated with high concentrations of ZA. The addition of GGOH had increased the expression of Rap1A up to a certain limit. The experiments proved that ZA acts directly on the mevalonate pathway and protein prenylation and that GGOH could be applied as a future local therapy to MRONJ.

## 1. Introduction

Bisphosphonates (BPs) are considered the keystone to treat bone disorders as osteoporosis, osteogenesis imperfecta, and Paget's disease as well as bone metastases from various malignancies as multiple myeloma or breast/prostate cancer. Despite the great benefits of BPs, medication-related osteonecrosis of the jaw (MRONJ) arouse as a potential side effect of two pharmacological agents: antiresorptives (including bisphosphonates (BPs) and receptor activator of nuclear factor kappa-B ligand inhibitors) and antiangiogenics. MRONJ pathogenesis has been widely investigated, yet not fully understood. Lately, various factors have been formulated to discuss the possible mechanism as interaction between bone turnover, impairment of angiogenesis, infection, local trauma, oral mucosal toxicity, or immunomodulation [1–3]. However, the most accepted theories being the influence of BPs on angiogenesis or cessation of bone remodelling and turnover by suppressing osteoclast and osteoblast activity leading to areas of necrotic bone [4]. Recently, bacterial infection to the maxillofacial region has been suggested as a key factor for the pathogenesis and progression of MRONJ [5, 6].

BPs are stable analogues of natural inorganic pyrophosphates [7] broadly classified into two major classes with different mechanisms of action: nonnitrogen-containing BPs (NN-BPs) acting by incorporation into ATP and nitrogen-containing BPs (N-BPs) acting by inhibiting farnesyl diphosphate synthase (FDPS) in the mevalonate pathway (MVP) with zoledronate (ZA) being the most potent [8]. Inhibition of farnesyl diphosphate synthase prevents the synthesis of farnesyl diphosphate (FPP) and its derivative, geranylgeranyl diphosphate (GGPP) [9].

At the molecular level, ZA inhibits specific enzymes of the MVP resulting in the loss of isoprenoid intermediates altering protein prenylation which is required for the posttranslational maturation of the small GTP-binding proteins which are divided into at least five families, including Ras, Rho, Rab, Arf, and Ran [10]. The inhibition of these small GTPases plays a critical role in cellular growth and differentiation, cytoskeletal reorganisation, gene expression, and membrane ruffling interfering with osteoblast function resulting in impaired osteogenesis together with inducing apoptosis in osteoclast due to the disruption of the cytoskeleton and resorptive activity [11, 12].

Isoprenoid compounds as farnesol (FOH) and geranylgeraniol (GGOH) are intermediate products in the MVP essential for cell proliferation [13]. GGOH was developed in Japan being used orally as an antiulcer drug protecting the gastric mucosa from stress without affecting the gastric acid secretion [14]. It has positive effects on different cell lines treated with BPs by salvaging protein isoprenylation improving cell viability, proliferation, and migration in tissue regeneration thus overcoming N-BP-induced apoptosis [15, 16]. Some studies had supported the use of GGOH in angiogenesis theory [17] and local toxicity theory [18]. However, this study had supported the bone turnover theory with the use of GGOH.

Thus, the aims of this study were to (1) investigate the effect of different concentrations of ZA on the bone cells and (2) understand if isoprenoids as GGOH was able to rescue bone cells which could be proposed as a future local therapy for the treatment of MRONJ.

## 2. Materials and Methods

### 2.1. Culture of the Cells

*Human osteoblasts (hOBs)* were purchased from Sigma Aldrich (Cat no. 406-05A, Munich, Germany) and were always cultured at a density of 3.5 × 10^4^ on a 35 mm Petri dish in osteoblast growth medium (Cat no. 417500) at 37°C in a humidified atmosphere of 5% CO_2_. The medium was changed twice per week and cells were subcultured when they reached 90% confluency. Cells between passages 3 and 6 were used from two different donors for the experiments.

*Human osteoclast precursors (hOCs)* and culture media were purchased from Lonza (Cat no. 2T-110, Basel, Switzerland). To induce osteoclast differentiation, cells were seeded in 6-well plates at a density of 1.0 × 10^4^ cells/well and cultured in complete culture medium supplemented with 25 ng/mL macrophage colony-stimulating factor (M-CSF; PeproTech, Hamburg, Germany) and 50 ng/mL receptor activator of NF-*κ*B ligand (RANKL; PeproTech, Hamburg, Germany). Cells were maintained in a humidified atmosphere containing 5% CO_2_ at 37°C with medium change twice per week until the cells were used for the experiments. Cells at passage 1 were used for all the experiments as OCs could not be subcultured. Osteoclasts were always seeded at a density of 1.0 × 10^4^ cells/well in 6-well plates for WST-1 assay, live/dead assay, and TRAP staining.

### 2.2. Preparation of Drugs

#### 2.2.1. Mevalonate Pathway Inhibitors

Zoledronate (ZA) obtained as a gift from Chemos (Regenstauf, Germany) was chosen due to its high relative potency increasing the probability of osteonecrosis. A stock solution of 20 mM ZA was prepared by dissolving the powder in physiological saline (0.9% NaCl), sterile filtered before use, and stored at -20°C. The drug was diluted in appropriate culture media to the given concentration to be used in the experiments. The dose range was chosen to be 0.1, 25, and 100 *μ*M of ZA.

#### 2.2.2. Mevalonate Pathway Activators

Geranylgeraniol (GGOH) was purchased from Sigma (G3278-100MG; Munich, Germany). A stock solution of 5 mM was prepared by dissolving GGOH in pure ethanol, sterile-filtered, and stored at -20°C. Different concentrations of GGOH (10, 20, 40, and 80 *μ*M) were used for the experiments in an attempt to rescue the inhibitory effects of ZA.

### 2.3. Cell Treatment

Cells were seeded at the required density in the cell culture plates and incubated before treatment for 24 hours. For use in experiments, different concentrations of GGOH and ZA were diluted in the culture media and used throughout the whole experiment. The drugs were administered individually or simultaneously in combination for 7 days. Cells cultured without ZA/GGOH drugs served as the negative control while cells cultured with ZA or GGOH served as positive controls.

### 2.4. Viability of Bone Cells by WST-1

To determine whether ZA, GGOH, or ZA/GGOH affected cell growth in culture, cellular proliferation experiments were performed via WST-1 assay kit (Roche Diagnostics, Mannheim, Germany) according to the manufacturer's instructions on hOBs and hOCs. In brief, cells were seeded at the required density in complete culture medium for 24 h. Next day, the cells were treated with ZA, GGOH, or ZA/GGOH for 7 days. For measurements, the medium was replaced by fresh medium supplemented with WST-1 reagent added directly into the incubation media (diluted 1 : 10 with culture media). After 4 hours of incubating the cells at 37°C in 5% CO_2_ to form purple formazan crystals, the optical absorbance of the supernatants was determined at 450 nm against a reference wavelength of 620 nm using Multiskan FC microplate reader (Thermo Scientific, Massachusetts, USA). All WST-1 experiments were performed in triplicate and repeated at least 3 times to obtain the mean values. Cytotoxicity of the compounds was expressed as percentage cell viability compared to control. The absorbance of cells exposed to normal culture media (negative controls) was considered to be 100% cell viability. Positive controls were reading obtained from cells treated with ZA or GGOH.

### 2.5. Live/Dead Staining

Live/dead staining was used to analyse qualitative cell viability by staining the cells with Calcein-AM/EthD-III (Live/Dead fluorescent Cell Staining Kit II, PromoKine, PK-CA707-30002, PromoCell GmbH, Heidelberg, Germany) according to the manufacturer's protocol. In brief, cells were cultured for 24 h to allow attachment then treated with ZA or ZA/GGOH for 7 days. The cells were washed twice with PBS and sufficient volume of Calcein-AM/EthD-III staining solution was added to cover the cell monolayer. The cells were incubated for 30-45 minutes at room temperature protected from light then observed under the fluorescence microscope (AxioObserver Z1; Zeiss, Oberkochen, Germany). The experiment was repeated three times from two different donors.

### 2.6. Tartrate-Resistant Acid Phosphatase (TRAP) Staining

Osteoclasts (OCs) were treated with different concentrations of zoledronate (ZA) as well as GGOH for 7 days and incubated at 37°C in 5% CO_2_. To evaluate osteoclast formation in all groups, tartrate-resistant acid phosphatase (TRAP) staining was performed using Acid Phosphatase, Leukocyte (TRAP) Kit (Cat no.387A-1KT, Sigma Aldrich, Munich, Germany) according to the manufacturer's instructions.

In brief, OCs were washed with phosphate buffered solution (PBS), fixed by combining 3 parts of citrate solution, 8 parts of acetone, and 1 part of 37% formaldehyde for 30 seconds at 25°C and fixed cells were washed three times with PBS. During this time, the commercially available TRAP stain was prepared by prewarming to 37°C and added to each well of the plate to be stained. The plates were placed in the water bath at 37°C for 1 h protected from light. Later, the TRAP stain was aspirated and the wells were washed 3 times with prewarmed deionized water. The wells were counterstained with Gill's Hematoxylin for 1–2 min. The wells were washed with alkaline tap water until an adequate colour intensity of the stain is achieved, typically when nuclei appear blue. TRAP-positive multinucleated cells (>3 nuclei) were observed and counted under an Axiovert 40 CFL microscope (Zeiss, Oberkochen, Germany). The positive cells developed red colour of different intensity.

### 2.7. Protein Isolation in OBs and OCs

For protein analysis, hOBs and hOCs were seeded at the required density and were let to adhere in the 6-well plates for 24 h. Next day, the cells were treated with different concentrations of ZA or ZA/GGOH for 7 days. After treatment, the cells were washed with ice-cold PBS and lysed in 500 *μ*L of radioimmunoassay precipitation buffer (RIPA) containing 1x protease inhibitors (Complete protease inhibitor cocktail tablets, Roche, Mannheim, Germany) and then cleared by centrifugation at 10,000g for 10 min at 4°C. The protein content was quantified from the supernatant using protein quantification assay.

### 2.8. BCA Protein Assay

The Micro BCA Protein Assay Kit (Cat no.23235, Thermo Scientific, Massachusetts, USA) was used according to the manufacturer's instructions. Bovine serum albumin (BSA, 2 mg/mL) was used to create the standard curve. Absorbance was measured at 562 nm on Multiskan FC microplate reader (Thermo Scientific, Massachusetts, USA). The amount of protein in each well was calculated by plotting against a standard curve.

### 2.9. Western Blot of Rap1A

Ten microgram (10 *μ*g) aliquots of cleared protein extracts from hOBs and hOCs lysed cell samples were separated on a 15% sodium dodecyl sulphate-polyacrylamide (SDS-PAGE) gel. Proteins were transferred onto polyvinylidene difluoride membrane (PVDF membrane, BioRad, California, USA) using wet electroblotting. The membranes were incubated overnight at 4°C with the primary antibodies after blocking with 5% skimmed milk in 0.1% Tween-20 tris-buffered saline solution (TBST). The primary antibodies and their dilutions were as follows: rabbit anti-human Rap1A/B (1 : 500 dilution, VPA00481, BioRad, Munich, Germany) and mouse anti-human GAPDH (1 : 400 dilution, MAB5718, R&D Systems, Munich, Germany). The primary antibodies were followed by incubation with secondary antibodies: goat anti-rabbit horseradish peroxidase-conjugated (HRP-conjugated, Cat no. 7074P2, Cell Signaling Technology, Frankfurt am Main, Germany) or goat anti-mouse (Cat no. 610-1316, Rockland, Limerick, USA) at room temperature for 1 h. Membranes were developed with the enhanced chemiluminescence ImageQuant LAS 4000 mini system (GE Healthcare Life Sciences, Freiburg, Germany). Proteins were detected using Luminata Forte Western HRP Substrate (Millipore, Darmstadt, Germany). Luminescence intensities were quantified with ImageJ (http://imagej.nih.gov/ij/). The blots were striped and rehybridized. Data was normalized to GAPDH band densities as loading control.

### 2.10. Statistical Analysis

All experiments were performed in triplicates from two different cell lots and the results were presented as the mean ± standard deviation. The data were analysed by two-way analysis of variance (ANOVA) with Bonferroni posttests for absolute data between the positive control and treated cells and levels of statistical significance were indicated at ^∗^*P* < 0.05, ^∗∗^*P* < 0.01, ^∗∗∗^*P* < 0.001, and ^∗∗∗∗^*P* < 0.0001. Statistical analysis was performed using GraphPad Prism version 5.00 for Windows (GraphPad Software, San Diego California USA, https://www.graphpad.com/).

## 3. Results and Discussion

### 3.1. Results

#### 3.1.1. Viability of Bone Cells by WST-1

**Figure 1:**
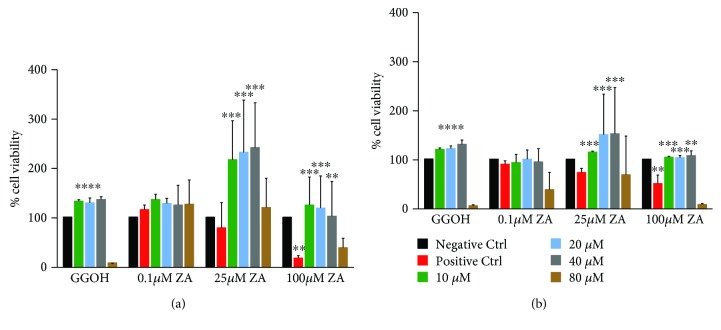
WST-1 activity of bone cells (OBs and OCs). Effect of ZA (0.1, 25, and 100 *μ*M) and GGOH (10, 20, 40, and 80 *μ*M) on the cell activity of bone cells. The cell proliferation was measured by WST-1 assay. (a) Human osteoblasts and (b) human osteoclasts were treated with different concentrations of ZA and GGOH for 7 days. The results are presented as the mean and standard deviation of the percentage of cell viability to the positive controls (*n* = 6). The results represent absorbance values measured at 450 nm against the background control wells using 650 nm as a reference. ANOVA tests and Bonferroni corrections for multiple comparisons were applied. Significant difference between controls and test groups: ^∗^*P* < 0.05, ^∗∗^*P* < 0.01, ^∗∗∗^*P* < 0.001, and ^∗∗∗∗^*P* < 0.0001.

The effect of GGOH on rescuing cells treated with ZA was studied and the results of WST-1 assay are presented in Figure 1. The cell viability was compared to the positive controls which were cells cultured in complete culture media treated with different concentrations of ZA or GGOH.

The viability of the cells was considered to be 100% in all the control groups after 7 days in the osteoblasts and osteoclasts.

In human osteoblasts, the treatment of the cells with low to moderate concentration of GGOH alone (10, 20, and 40 *μ*M) had enhanced the cell viability to be about 150% but higher concentration of GGOH (80 *μ*M) had decreased the viability significantly to be about 2% (*P* < 0.0001). The lowest dose of ZA (0.1 *μ*M) did not significantly affect the cell viability; instead, it tended even to slightly stimulate cell growth in hOBs to be 120%. At 25 *μ*M ZA, the cell viability was decreased to be about 70-80%. However, very high concentration of ZA (100 *μ*M) had significantly decreased the cell viability to be 20% (*P* < 0.01) which proves the cytotoxic effect of ZA on osteoblasts leading to their death. GGOH was the rescue agent that reversed the action of ZA on the cells. The addition of GGOH at different concentrations resulted in reversing the negative effect of ZA by means of increasing the cell viability in a positive manner (*P* < 0.001) (Figure 1(a)).

In human osteoclasts, the treatment of the cells with low to moderate concentration of GGOH alone (10, 20, and 40 *μ*M) had enhanced the cell viability to be about 110-150% but higher concentration of GGOH (80 *μ*M) had decreased the viability significantly to be about 1% (*P* < 0.0001). The cell viability had slightly decreased to be about 90% at 0.1 *μ*M ZA, while at 25 *μ*M ZA, the cell viability decreased to be about 75%. At very high concentration of ZA (100 *μ*M), the cell viability had significantly decreased to be 45% (*P* < 0.01) while the addition of GGOH at low and moderate concentrations (10-40 *μ*M) resulted in rescuing the cells and affected cell viability in a positive manner. In contrast, the addition of very high concentration of GGOH (80 *μ*M) resulted in affecting the cells in a negative manner decreasing the cell viability compared to the positive control group (Figure 1(b)). From our results, GGOH had a detectable effect on cell viability fully antagonizing the inhibition of cell growth induced by ZA. However, at higher concentrations, it had increased the effect of ZA. These findings suggested that GGOH had rescued the cell viability up to a certain limit. At lower concentrations, it had an antagonistic effect to ZA. However, at higher concentrations, it had enhanced the cell death.

#### 3.1.2. Live/Dead Staining

**Figure 2:**
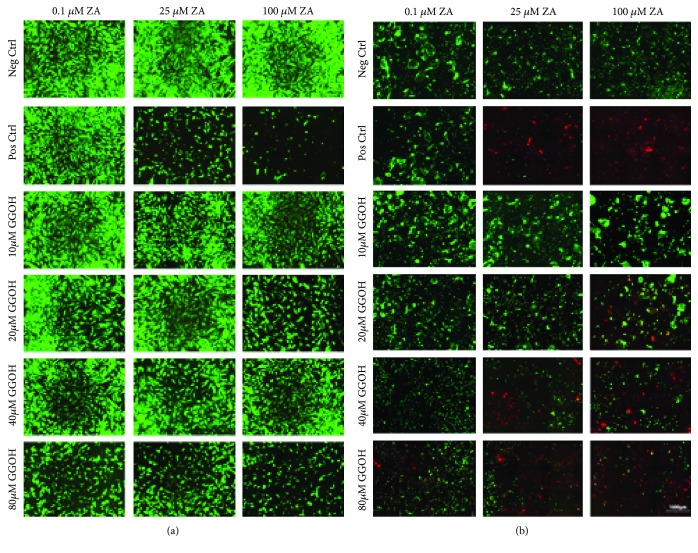
Live/dead staining of bone cells (OBs and OCs). Fluorescence microscopy of live and dead cells treated at different concentrations of ZA (0.1, 25, and 100 *μ*M) and GGOH (10, 20, 40, and 80 *μ*M) on (a) human osteoblasts and (b) human osteoclasts after 7 days examined with fluorescence microscope. Magnification: ×10, scale bar: 1000 *μ*m. The experiments were performed in triplicates from two different cell lots. Living cells were detected as green fluorescence and dead cells were detected as red fluorescence.

Live/dead staining was performed after 7 days in order to test whether the apoptosis of bone cells by ZA was reversed by the mevalonate pathway metabolite (GGOH). The fluorescent microscopic analysis of live/dead cells is shown in Figure 2, where living cells were presented as green and dead cells were red.

As shown in Figures 2(a) and 2(b), bone cells cultured at the normal complete culture media (negative controls) were completely living expressed as green cells. However, following the exposure of the cells to different concentration of ZA had resulted in variable effects. At the concentration of 0.1 *μ*M ZA alone, both cell lines were viable while at 25 *μ*M ZA, osteoblasts were viable with decreased density and loss of a lot of cells; as for the osteoclasts, most of the cells were dead with very few viable cells. At the 100 *μ*M ZA, the cell density was even less than that of the 25 *μ*M for the osteoblasts. However, for the osteoclasts, the cells were completely dead expressed as red cells. This proves that high concentrations of ZA (25 and 100 *μ*M ZA) had cytotoxic effects on bone cells.

The simultaneous addition of lower concentrations of GGOH had reversed the negative effect of ZA on bone cells. The addition of 10 and 20 *μ*M GGOH had increased the viability of cells compared to the positive controls. The concentration of 40 *μ*M was a borderline concentration as it had increased the cell viability in osteoblasts while it decreased the cell viability in osteoclasts. At very high concentration of GGOH (80 *μ*M), it had decreased the viability of the osteoclasts with special concern to the 25 and 100 *μ*M ZA expressed as red cells. However, it had decreased the cell density of the cells but not causing their complete death. At high concentration of ZA (100 *μ*M) and GGOH (80 *μ*M), the cell densities were decreased significantly.

#### 3.1.3. Tartrate-Resistant Acid Phosphatase (TRAP) Staining

The OCs were treated with ZA alone or a combination of ZA and GGOH for 7 days.

**Figure 3:**
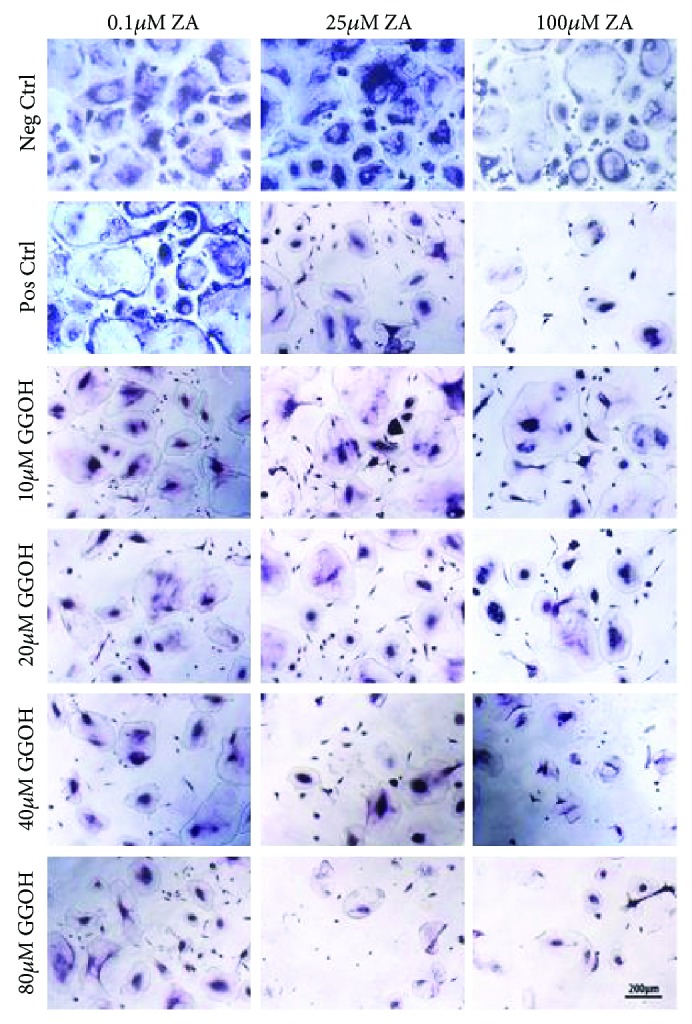
TRAP staining assay of osteoclast. Osteoclasts were cultured at different concentrations of ZA (0.1, 25, and 100 *μ*M) and GGOH (10, 20, 40, and 80 *μ*M) for 7 days. Magnification: ×10, scale bar: 200 *μ*m. TRAP staining of human osteoclasts differentiated in vitro on polystyrene flasks. Representative images are depicted from two different cell lots.

As demonstrated in Figure 3, multinucleated osteoclasts in large numbers stained positive in negative controls. Oppositely, ZA significantly influenced osteoclasts in a concentration-dependent manner as evidenced by TRAP staining analysis. The concentration of 0.1 *μ*M ZA did not affect the proliferation and the differentiation of OCs. However, at the concentration of 25 *μ*M ZA, the differentiation was reduced. Furthermore, 100 *μ*M ZA exhibited maximum inhibitory effect on osteoclast generation as evidenced by counting the number of TRAP-positive osteoclasts.

In contrast, multinucleated osteoclasts expressing TRAP were detected in ZA cultures combined with low concentration of GGOH (10 *μ*M). Higher concentrations of GGOH (20 and 40 *μ*M) reversed the action of ZA in the positive controls but less than that of 10 *μ*M. GGOH increased significantly the amount of TRAP enzyme when compared with ZA-treated cells (positive control) up to a certain limit. TRAP staining confirmed the results obtained from the live/dead assay and WST-1. At very high concentration of GGOH (80 *μ*M), the osteoclasts were apoptotic expressing less TRAP staining with reduced number of cells.

#### 3.1.4. Immunoblotting for Protein Prenylation in OBs and OCs

**Figure 4:**
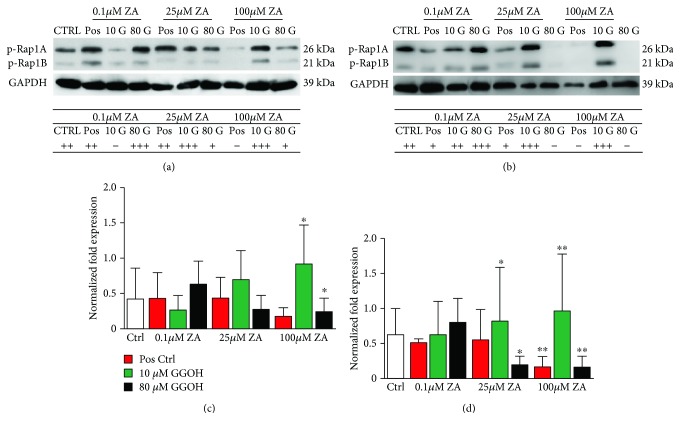
Western blot with anti-Rap1A/B antibody in bone cells (OBs and OCs). Effect of ZA (0.1, 25, and 100 *μ*M) and GGOH (10, 20, 40, and 80 *μ*M) on protein prenylation of (a) human osteoblasts and (b) human osteoclasts after 7 days. GAPDH was used as loading control for western. The results are expressed as fold change of p-Rap1A/B (prenylated Rap1A/B). The results are presented as the mean and standard deviation (*n* = 6). ANOVA tests and Bonferroni corrections for multiple comparisons were applied. Significant difference between controls and test groups: ^∗^*P* < 0.05, ^∗∗^*P* < 0.01.

Immunoblot analysis was performed in order to confirm the impairment of geranylgeranylation by ZA in bone cells and to understand the mechanism of GGOH rescue of cellular viability and metabolic activity in proliferating cells. The prenylation status of Rap1A/B, which is a member of the GTPase superfamily of proteins known to modulate cellular activity, is shown in Figure 4.

In hOBs, Rap1A/B was detected in the negative controls, positive controls of ZA (0.1 and 25 *μ*M) but was absent in cells treated with high concentrations of ZA (100 *μ*M). The addition of GGOH had a different effect on hOBs. At the concentration of 0.1 *μ*M ZA, 10 *μ*M GGOH had a negative effect on the Rap1A/B expression. However, the same concentration of GGOH (10 *μ*M) resulted in increased expression of Rap1A/B in combination with 25 or 100 *μ*M ZA. High concentration of GGOH (80 *μ*M) resulted in decreased expression of Rap1A/B in both 25 and 100 *μ*M ZA proving the effect of ZA and GGOH on the protein prenylation and on the mevalonate pathway. What was different is that the combination of low concentration of ZA (0.1 *μ*M) and high concentration of GGOH (80 *μ*M) had increased the expression of Rap1A/B. Our results proved the effect of ZA and GGOH on protein prenylation and the mevalonate pathway although the very low concentration of ZA was somehow misleading giving different results than that of moderate or high concentrations of ZA (Figure 4(a)). In hOCs, the effect of ZA or GGOH was clearly detected. Rap1A/B was expressed in the negative control and more effectively by the addition of 10 *μ*M GGOH to the 25 and 100 *μ*M ZA cell cultures. However, its expression was so much reduced or even absent at the positive controls which proves that ZA acts directly on the mevalonate pathway and protein prenylation. The addition of 80 *μ*M GGOH to the cells already treated with ZA was different at different concentrations. At the concentration of 0.1 *μ*M, Rap1A/B was more expressed in combination of that high concentration of GGOH. In contrast, at the concentration of 25 and 100 *μ*M, Rap1A/B was not expressed in osteoclasts proving that GGOH at high concentration was synergistic to ZA and completely inhibited the mevalonate pathway (Figure 4(b)).

## 4. Discussion

The aim of this study was to reveal the great role of GGOH as mevalonic acid metabolite on reversing the profound cytotoxic effect of zoledronate (N-BP) on the function of bone cells suggesting that it could be a future local therapy for the treatment of medication-related osteonecrosis of the jaw (MRONJ). Moreover, we had shown that ZA induced cessation of mevalonate pathway and stopped protein prenylation and consequently induced cell death.

MRONJ is a well-known serious complication of antiresorptive therapy with denosumab or N-BPs [19]. Those N-BPs are potent inhibitors of the mevalonate pathway which is responsible for the production of cholesterol and isoprenoid such as vitamin D and steroid hormones. Several enzymes along the mevalonate pathway have been studied as potential molecular targets for N-BPs causing inhibition of the synthesis of the prenylation of small GTP-binding proteins such as Ras, Rho, Rac, Rab, and Cdc42 regulating osteoclast morphology, cytoskeleton arrangement, membrane ruffling, trafficking, and cell survival [20].

Zoledronic acid (ZA) is the strongest inhibitor of farnesyl pyrophosphate synthase compared to other N-BPs [21] used in our experiments due to its highest potency and being the most commonly used intravenous BP with the longest duration of action, while at the same time it is associated with the highest risk of developing MRONJ [22].

In the present study, it was observed that high concentrations of ZA resulted in significant decrease in the metabolic activity and cell viability of bone cells. However, low concentrations of ZA appeared to have no effect on metabolic activity or cell viability. This was consistent with previous studies [23, 24]. Although ZA acts mainly on osteoclasts, it may also target other cell types such as osteoblasts in which ZA preserved the viability of osteoblasts inhibiting their apoptosis [25] or even very low concentrations of ZA stimulate osteogenic differentiation and survival of MSCs [26–28]. It has also been pointed out that higher concentrations of N-BPs are likely to be necessary for intracellular inhibition of small GTPase prenylation in osteoblasts [29].

Geranylgeraniol (GGOH) is an isoprenoid playing different roles in various physiological processes in animals and plants. It was selected according to its reliance on mevalonate input where it has the ability not only to improve the side effects of bisphosphonate therapy by regulating the mevalonate pathway but also acts as anti-inflammatory, antitumorigenic, and neuroprotective [30].

The management of MRONJ is controversial with no current gold standard treatment. Several local treatment options have been described starting with local application of antibiotics, surgical debridement or hyperbaric oxygen (HBO) therapy, and using growth and differentiation factors [31]. However, recently, using cytoprotectant agents as GGOH is a new and promising approach in MRONJ management [32].

Several studies have revealed the effects of increased viability and migration that GGOH caused in different cells previously treated with ZA. These cells included oral fibroblasts [33], human umbilical endothelial cells [34], and oral keratinocytes [18].

Although GGOH reverses the effects of BPs in the mevalonate pathway, it acts as a double weapon in the treatment of MRONJ in which the systemic administration would lead to faster and easier transport of GGOH to the cells, especially to the basal mucosal layers. However, systemic administration of GGOH may be problematic as it decreases the pharmacological action of the BPs with special concern to malignant patients facing the risk of the spread of the malignancy and high morbidity rate. To overcome these complications, it would be of great benefit to apply GGOH locally [22]. This could be achieved through mouth rinses which seem to be a promising application route due to the localization of oral keratinocytes in the mucosa or during surgical procedures with other local drug delivery systems such as collagen membranes [34]. However, further in vivo studies should be performed to ascertain dosage, safety, and efficacy of GGOH.

Protein prenylation is mediated by three protein prenyltransferase enzymes: farnesyltransferase, for farnesylation of proteins such as Ras and nuclear lamins; geranylgeranyltransferase type I, for geranylgeranylation of proteins such as Rho, Rac, and Rap1 (Ras-associated protein); and geranylgeranyltransferase type II, for geranylgeranylation of Rab [35].

Ras-associated protein (Rap) is the prototype for a large superfamily of GTPases belonging to the Ras superfamily that regulates multiple cellular processes and includes 5 members, Rap1A, Rap1B, Rap2A, Rap2B , and Rap2C, which are grouped into 2 subfamilies, Rap1 and Rap2, based on their sequence homology. Rap1A and Rap1B were shown to be essential for integrin activation and cell-cell adhesion of various cell types, such as leukocytes, platelets, fibroblasts, and progenitor cells [36]. Rap1A has an essential role in regulating osteoclast function. However, the exact role and the underlying mechanism mediated by Rap1A to regulate osteoblastic differentiation are unclear [37].

In our experiments, we have examined the expression of Rap1A proteins not only in osteoclasts but also in osteoblasts and found that the levels of Rap1A expression were also changed according to the conditions of our experiments in osteoblast cultures indicating that Rap1A plays a role in these cell lines. Rap1A was used as a convenient biomarker for the impairment of posttranslational modification by geranylgeranylation of proteins in tissues. As determined by the characterization of Rap1A, ZA inhibited the mevalonate pathway and consequently inhibited the protein prenylation in bone cells in a dose-dependent manner. The combination of GGOH with ZA has been reported to reverse the inhibitory effects of ZA in some cell lines [38]. In the present study, we have found that the addition of GGOH, which is converted to geranylgeranyl pyrophosphate (GGPP), restored geranylgeranylation of Rap1A. Our results demonstrated that the mevalonate pathway is involved in osteoclast-mediated bone resorption and suggest that nitrogen-containing bisphosphonates affect mainly protein geranylgeranylation.

A new finding in our experiments was that ZA not only has shown potential for synergistic interaction with GGOH at very high concentrations of both drugs inducing apoptosis and cell death but also that GGOH alone is cytotoxic at very high concentrations. The protective role of GGOH is not generalized to all cell types. In some studies, GGOH did not protect rat hepatocytes from apoptosis and was toxic [39], increased DNA fragmentation in cells exposed to GGOH at concentrations higher than 50 *μ*M [40]. It is likely that with the above certain concentrations and incubation times, GGOH could be proapoptotic.

## 5. Conclusions

Although GGOH had a detectable effect on cell viability fully antagonizing the inhibition of cell growth induced by ZA, higher concentration of GGOH had enhanced the effects of ZA. GGOH had rescued the bone cells by acting on the protein prenylation. Thus, GGOH could be applied as a future local therapy to MRONJ. However, more experiments are required to be performed on GGOH in in vivo animal models to ensure its positive effects.

## Data Availability

The data used to support the findings of this study are available from the corresponding author upon request.
